# Impact of amyloid-beta changes on cognitive outcomes in Alzheimer’s disease: analysis of clinical trials using a quantitative systems pharmacology model

**DOI:** 10.1186/s13195-018-0343-5

**Published:** 2018-02-02

**Authors:** Hugo Geerts, Athan Spiros, Patrick Roberts

**Affiliations:** 1 In Silico Biosciences, 686 Westwind Dr, Berwyn, PA 1312 USA; 2 Perelman School of Medicine, University of Pennsylvania, Philadelphia, PA USA; 3 Amazon AI AWS, Portland, OR USA

**Keywords:** Amyloid secretase inhibition, Biologics, Amyloid load, Modeling, Trial failure, Patient selection, Prevention trials

## Abstract

**Background:**

Despite a tremendous amount of information on the role of amyloid in Alzheimer’s disease (AD), almost all clinical trials testing this hypothesis have failed to generate clinically relevant cognitive effects.

**Methods:**

We present an advanced mechanism-based and biophysically realistic quantitative systems pharmacology computer model of an Alzheimer-type neuronal cortical network that has been calibrated with Alzheimer Disease Assessment Scale, cognitive subscale (ADAS-Cog) readouts from historical clinical trials and simulated the differential impact of amyloid-beta (Aβ40 and Aβ42) oligomers on glutamate and nicotinic neurotransmission.

**Results:**

Preclinical data suggest a beneficial effect of shorter Aβ forms within a limited dose range. Such a beneficial effect of Aβ40 on glutamate neurotransmission in human patients is absolutely necessary to reproduce clinical data on the ADAS-Cog in minimal cognitive impairment (MCI) patients with and without amyloid load, the effect of APOE genotype effect on the slope of the cognitive trajectory over time in placebo AD patients and higher sensitivity to cholinergic manipulation with scopolamine associated with higher Aβ in MCI subjects. We further derive a relationship between units of Aβ load in our model and the standard uptake value ratio from amyloid imaging.

When introducing the documented clinical pharmacodynamic effects on Aβ levels for various amyloid-related clinical interventions in patients with low Aβ baseline, the platform predicts an overall significant worsening for passive vaccination with solanezumab, beta-secretase inhibitor verubecestat and gamma-secretase inhibitor semagacestat. In contrast, all three interventions improved cognition in subjects with moderate to high baseline Aβ levels, with verubecestat anticipated to have the greatest effect (around ADAS-Cog value 1.5 points), solanezumab the lowest (0.8 ADAS-Cog value points) and semagacestat in between. This could explain the success of many amyloid interventions in transgene animals with an artificial high level of Aβ, but not in AD patients with a large variability of amyloid loads.

**Conclusions:**

If these predictions are confirmed in post-hoc analyses of failed clinical amyloid-modulating trials, one should question the rationale behind testing these interventions in early and prodromal subjects with low or zero amyloid load.

## Background

Amyloid-beta (Aβ) has been proposed as a key neuropathological contributor in Alzheimer’s disease (AD) [1] and modulation of amyloid load in Alzheimer patients has been the focus of many clinical trials, but with mixed results [2]. However, clear insights into the dynamics of various Aβ monomeric, oligomeric and aggregated forms, their impact on cognitive readouts and the effect of various therapeutic interventions with amyloid antibodies on the distribution of different forms in human patients are lacking. In addition, transgene animal models often use nonrealistic Aβ levels in order to amplify the pathology and improve identification of therapeutic drug interventions.

Recent stable isotope labeling kinetics (SILK) studies in humans have allowed one to numerically constrain the synthesis rates of Aβ and kinetic models have been developed to account for these observations [3, 4]. These results confirm the relatively greater level of the shorter Aβ40 over the longer Aβ42 form and suggest that the human brain cares less about restricting Aβ40 levels. In addition, a number of preclinical studies suggest a different role for short versus long Aβ forms. Notably, evidence has been presented for the shorter Aβ40 form having a beneficial neurostimulatory effect on synaptic function [5–7]. An important question is whether a similar process is active in the human AD brain. Given that the oligomeric Aβ40 and Aβ42 peptides are probably the species that differentially affect synaptic function [8], how do changes in both Aβ levels impact clinically relevant cognitive outcomes as measured by the ADAS-Cog?

In this paper, we propose to address these questions using a mechanism-based computer simulation of complex networks. This approach called quantitative systems pharmacology [9] (QSP) has been applied in other indications, such as cardiovascular disorders, metabolic disorders and inflammation [10] and in the CNS [11–13], and is increasingly being studied for its possible applications in regulatory decisions [14]. Basically such an approach allows one to combine a wide variety of different datasets with a formalized form of domain expertise, essentially generating parts of ‘virtual’ human patients and partially mitigating translational issues associated with rodent models [15].

We use a neuronal network computer model that incorporates the biological effects of these two peptide forms on glutamate neurotransmission and the nicotinic α7 receptor (nAChR) in order to generate an output that is proportional to a cognitive scale such as the ADAS-Cog. This ADAS-Cog calibrated model has been described before [16] and has been able to correctly predict an unexpected clinical outcome for a novel candidate Alzheimer drug [12]. Such a model would be amenable to be constrained by clinical data, as the predicted cognitive outcomes can be compared to the observed clinical effects.

As with any modeling approach, we will constrain the parameters such that the QSP model can reproduce clinical datasets. We will focus on the following clinical datasets: the absolute ADAS-Cog values for minimal cognitive impairment (MCI) subjects with and without Aβ load [17]; the impact of Aβ on cholinergic dysfunction in MCI patients [18]; and the effect of differential Aβ clearance associated with the APOE genotype on the cognitive trajectory [19]. This will also allow us to calibrate the unit of amyloid load in the model using clinical data on the relation between amyloid imaging and cognitive status in MCI patients [17], and most importantly to derive ranges for the different parameters in the model that are consistent with the observed clinical datasets.

With these parameter values fixed, we will then study the impact of beta-secretase, gamma-secretase inhibition and passive vaccination of soluble amyloid peptides by solanezumab on a cognitive readout using their reported pharmacodynamic effects on various forms of Aβ in clinical trials.

## Methods

**Fig. 1:**
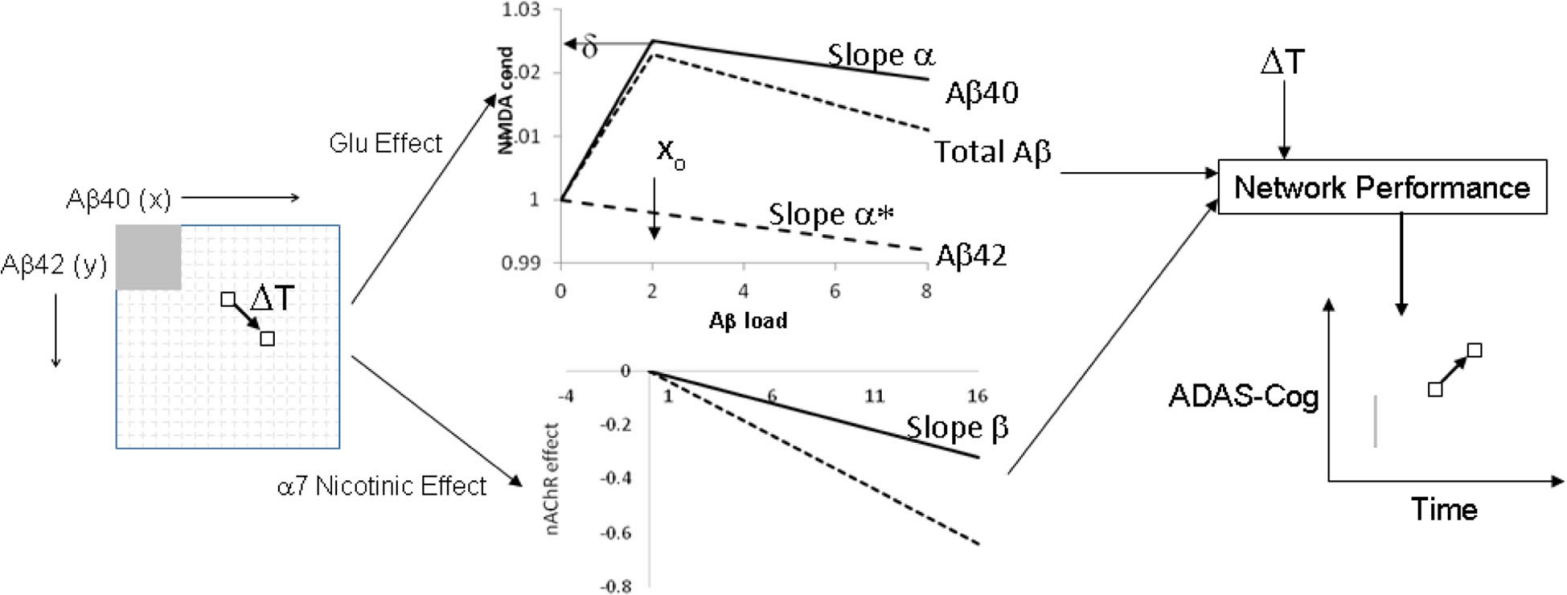
Modeling pipeline for simulating effect of changes in Aβ40 and Aβ42 concentrations on cognitive outcome. For each value of Aβ40 and Aβ42 (monomer, dimer and trimer) levels (left matrix), the effect on changes in excitatory–excitatory (e-e) NMDA conductance and α7 nAChR activation is derived from the coupling equations (top and bottom graphs, respectively) and the outcome on a cognitive neuronal network is calculated for the whole 17 × 17 matrix. Top figure shows the relationship between Aβ40 and Aβ42 load and e-e NMDA conductance with maximum value δ at position *x*_0_ and slopes α and α*. Bottom figure illustrates the relationship between Aβ load and α7 nAChR inactivation with slope β. We calculate the cognitive outcome matrix for a baseline value and for six different disease states (MCI, and mild-to-moderate AD patients at time 0, 12, 26, 52 and 78 weeks). Aβ load (left matrix) of an AD patient can be defined for any region, such as the one in gray which shows a possible low Aβ load case (the high Aβ load case would be everything outside the gray area) and its cognitive status can be determined by averaging the range of results at a particular time (the gray line in the right graph). Similarly, for any change in Aβ40 and Aβ42 low-order aggregates load as a consequence of disease pathology or therapeutic interventions over time span Δ*T* (going from one box to another box in the Aβ load matrix), corresponding changes in ADAS-Cog can be calculated based on their impact on cognitive readout changes using their glutamate (Glu) and α7 nAChR-mediated effects. Aβ amyloid-beta, ADAS-Cog Alzheimer Disease Assessment Scale, cognitive subscale, NMDA *N*-methyl-d-aspartate

The general modeling pipeline is illustrated in Fig. 1. The neurophysiological effects of various forms of Aβ40 and Aβ42 on glutamate and α7 nAChR neurotransmission are implemented (see later) in a humanized and ADAS-Cog calibrated cortical computer model as described before [12, 16].

Basically, the cortical computer model simulates the firing dynamics of a biophysically realistic multicompartment network of 80 prefrontal cortex glutamatergic pyramidal cells and 40 GABAergic interneurons, with full implementation of dopaminergic, serotonergic, noradrenergic and cholinergic modulation effects at their appropriate locations and coupled to their specific downstream voltage-gated ion channels. While this platform has been designed using in-vivo electrophysiological single-unit recordings in nonhuman primates [20] performing a working memory task, and therefore probably only reflects the maintenance phase of a working memory task, the outcome could be generalized to the strength of a memory trace [16].

Based on the level of Aβ40 and Aβ42 low-order aggregates (monomer, dimer and trimer), we implement a dose-dependent and isoform-dependent interaction on glutamatergic and nicotinic neurotransmission (Fig. 1). Low-dimension Aβ40 aggregates (monomer, dimer and trimer) have a complex effect on synaptic transmission [5, 21], with a stimulatory effect at low doses of Aβ40 and an inhibitory effect for high doses of Aβ40. Aβ42, on the other hand, decreases glutamatergic transmission monotonically over the whole dose range.

We implemented the glutamatergic effect of Aβ by modifying the maximum *N*-methyl-d-Aspartate (NMDA) conductance on excitatory to excitatory synapses mathematically as follows. We define *x* to be the Aβ40 load and *y* to be the Aβ42 load (monomer, dimer and trimer). We discretize the load size by allowing *x* and *y* to range from 0 to 16, a scale fine enough to see differences but coarse enough to be computationally efficient. With *x*_0_ being the Aβ40 low aggregate load corresponding to the maximal positive effect on NMDA conductance, we define the effect on NMDA conductance, *g*, with δ being the maximal relative effect as follows:

1A $$ g\left(x,y\right)={g}_0\cdot \left[1+\delta \left(x/{x}_0\right)-y{\alpha}^{\ast}\right]\kern0.5em \mathrm{if}\kern0.5em x\le {x}_0 $$

1B $$ g\left(x,y\right)={g}_0\cdot \left[1+\delta +\left({x}_0-x\right)\alpha -y{\alpha}^{\ast}\right]\kern0.5em \mathrm{if}\kern0.5em x>{x}_0 $$

Thus, the NMDA conductance continues to grow linearly with Aβ40 load, *x*, until *x* = *x*_0_ to a value of 1 + δ and then declines with slope α. NMDA conductance monotonically decreases with increasing Aβ42 load with slope α*. We assume *x*_0_ to be 2, so that we have a large dynamic range for Aβ modulated processes (but see Sensitivity analysis).

Similarly, when considering the effect of Aβ40 and Aβ42 load on α7 nAChR function, we define a coupling factor β (here the effects for the two Aβ forms are identical) so that:

2 $$ \alpha 7\ \left(x,y\right)\ \mathrm{activation}=\alpha 7\ \left(x,y\right)\ {\mathrm{activation}}_0\ast \left[1-\beta \left(x+y\right)\right]. $$

This results in a 17 × 17 matrix of values for the effect of both Aβ forms with changes in glutamate and on nicotinic neurotransmission as dimensions. We will restrict the values for δ, α, α* and β using clinical data (see Results).

We simulate the neuronal network outcome in the 17 × 17 matrix for each of the different disease states (corresponding to MCI, and AD at 0, 12, 26, 52 and 78 weeks trial duration; see [16]). For each (Aβ40, Aβ42) load (*x*, *y*), we then calculate the impact on both glutamatergic and nicotinic transmission using Equations (1A), (1B) and (2) to determine the result in the anticipated ADAS-Cog outcome (Fig. 1). The simulations are performed in NEURON Release 7.2 [22].

### Alzheimer pathology and amyloid deposition

AD pathology is introduced as a cholinergic deficit [23] of 30% in addition to a progressive loss of neurons and synapses [16], except for the case of MCI where a compensatory increase of 30% is used [24, 25]. Progressive neurodegeneration is simulated as a linear loss of neurons (at 0.35%/week) and synapses (0.04%/week), values that were constrained from historical clinical trials. We assume this neuronal degeneration is independent from Aβ deposition (see Discussion). In this study, amyloid deposition as a function of trial duration is arbitrarily set at 1 unit/13 weeks for both Aβ40 and Aβ42. As the concentration of Aβ40 is about 10-fold higher than Aβ42, the corresponding units are likely also 10-fold different. Although Aβ deposition is a balance between formation and clearance, this value assumes linear growth and helps to quantify the arbitrary units of Aβ load; that is, 1 unit is the amount of increased Aβ in a 13-week period for the APOE4^+/−^ heterozygote situation (see also the following).

### Calibration of the network

This ADAS-Cog model has been calibrated before [16] based on clinical trials with 28 different retrospective historical treatment outcomes in a mild-to-moderate AD population with MMSE between 18 and 24 and followed for 78 weeks, resulting in correlations (*R*^2^) above 0.6. The drug–dose combinations include placebo values from the flurbiprofen and tarenflurbil trial at 72 weeks, two doses and three time points for donepezil and rivastigmine, three doses and three time points for galantamine, and two doses and two time points for the 5-HT6 antagonist SB742457 for a population of mild-to-moderate AD patients. In addition, correlation coefficients at each individual time point (12, 26 and 52 weeks) are consistently above 0.50 and statistically significant at the *p* < 0.05 level [16].

Absolute baseline ADAS-Cog values are in the 20–22 range dependent upon the APOE genotype. For MCI patients, we have a similar relationship, but with an ADAS-Cog baseline value of 4.5 for healthy cognitively normal controls.

### Scopolamine

The effect of scopolamine on the relevant receptors in the working memory model, M_1_ mAChR and α_4_β_2_ and α_7_ nAChRs (indirect effect though the presynaptic M_2_ mAChR autoreceptor), is modeled in the receptor competition model [26, 27] using its affinity for M_1_ and M_2_ mAChR (*K*_*i*_ for M_1_ and M_2_ = 1.1 and 1.22 nM, respectively; data from the standardized PDSP database: https://pdspdb.unc.edu/pdspWeb/ [28]). Basically, this model simulates the competition between an endogenous neurotransmitter (here ACh) and scopolamine for postsynaptic cholinergic receptors, based on realistic firing frequencies to determine an average postsynaptic receptor activation that can be used in the working memory model. Note that M_2_ mAChR is implemented as a presynaptic autoreceptor with the appropriate negative feedback coupling on subsequent ACh release [29] (for more details see [16]) and that therefore scopolamine affects the activation state of both α_4_β_2_ and α_7_ nAChR with direct effects on Aβ (through its interaction on the same α_7_ nAChR receptor) and indirect effects by adjusting the excitation–inhibition balance (through α_4_β_2_ nAChR regulating GABA release).

### APOE genotype

We implement the effect of APOE4 in two ways: by adjusting the density of cortical synapses, by −20% for APOE4^+/+^ and by +20% for APOE4^−/−^ relative to the APOE4^+/−^ heterozygote genotype (for a review see [30, 31]); and by changing the deposition rate of Aβ to 1.50 units/13 weeks for APOE4^+/+^ and 0.50 units/13 weeks for APOE4^−/−^, a 50% difference compared to the deposition rates of the heterozygote APOE4^+/−^ form at 1 unit/13 weeks [31].

## Results

### Constraining system parameters using clinical data

The first major challenge is to obtain good estimates of the various parameters, notably *x*_0_, the position of the Aβ40 peak, δ, the magnitude of the Aβ40 beneficial effect, and α, α* and β, the slopes related to the reduction by both Aβ forms on Glu and nAChR neurotransmission, respectively, in addition to the ‘units’ of Aβ load. Ranges of parameter values must be able to reproduce clinically observed datasets. Once we set the relation between Aβ units and the standard uptake value ratio (SUVR) of amyloid imaging in the next section, we then simulate the outcome of two clinical experiments: the effect of scopolamine on cognition in MCI subjects with and without Aβ load [18]; and the effect of APOE genotype on cognitive trajectory in placebo-treated AD patients [19, 32].

To relate the Aβ levels (from 0 to 16 ‘units’) in the ADAS-Cog calibrated computer model to an experimentally determined value such as the SUVR, we studied the impact of Aβ on cognitive readout in an MCI model. Clinical data (see Table 1 in [17]) suggest that MCI patients with Aβ-positive load (average SUVR = 1.5 measured with florbetapir) have a baseline ADAS-Cog value of 10.8 vs 8.5 for subjects without Aβ load (average SUVR = 1.0). Normal elderly controls have an ADAS-Cog value of 5.6 and 4.1, respectively.

**Fig. 2:**
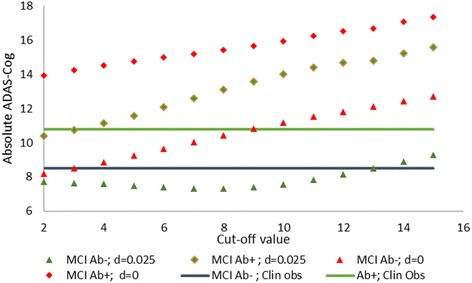
Absolute ADAS-Cog predictions in an MCI ‘virtual patient’ for different cutoff values for Aβ + and Aβ– imaging SUVR values. Reported ADAS-Cog values are 8.5 for MCI Aβ– patients and 10.8 for MCI Aβ + patients (blue and green horizontal lines, respectively). Cutoff values define the gray area in the Aβ load matrix of Fig. 1. For the optimal parameter set for δ, α, α* and β (*x*_0_ fixed at 2), the average ADAS-Cog prediction for Aβ– and Aβ + MCI subjects is shown by the green triangles and diamonds, respectively. At a cutoff value of 3, both predicted values (7.8 for Aβ– and 10.7 for Aβ + subjects) are near the clinical values. For the situation of δ = 0 (same values for α, α*, β and *x*_0_), the average ADAS-Cog prediction for Aβ– patients and Aβ + patients is shown by the red triangles and diamonds  (8.4 for Aβ– and 14.2 for Aβ + subjects, respectively). In this case, while the Aβ– MCI subjects cross the blue line in the correct range, the Aβ + MCI subjects result in an ADAS-Cog readout that is much greater than the Aβ + reported clinical values (green line). This suggests that the condition δ = 0 (no beneficial effect of Aβ40) is unable to reproduce this clinical outcome. Aβ amyloid-beta, A–β subjects with *x* ≤ 2 and *y* ≤ 2, Aβ + subjects with *x* > 2 and *y* > 2, ADAS-Cog Alzheimer Disease Assessment Scale, cognitive subscale, MCI minimal cognitive impairment

We simulate an MCI patient population in the cognitive model using a 3% decrease in synapse and neuron density in addition to a 30% increase in cholinergic tone [25]. We start from the value of 4.1 as the best performance of this computer model (in the absence of any Aβ load) and consider the average simulated outcome for a population within the 17 × 17 Aβ load matrix below and above a specific cutoff value that would result in the values for the ADAS-Cog mentioned earlier (see gray box in Fig. 1). For *x*_0_ = 2, δ = 0.015, α = 0.0015, α* = 0.00035 and β = 0.025, the average anticipated ADAS-Cog value in the QSP model for MCI patients amounts to 10.4 for all subjects with *x* > 2 and *y* > 2 (called Aβ+) and 7.4 for subjects with *x* ≤ 2 and *y* ≤ 2 (called Aβ–), the closest we can get to the measured 10.8 and 8.5 values respectively (see Fig. 2). This would fix the cutoff value between Aβ + and Aβ– load to be 3 units in our model.

With slopes α, α* and β constant, increasing δ also significantly increases the cutoff value in order for a difference of between 2 and 3 points for Aβ + vs Aβ– MCI subjects to occur. This makes sense as increasing δ with constant slope α necessitates a larger Aβ40 load that would give an inhibitory effect on Glu neurotransmission. This is equivalent to increasing the gray box in the Aβ load matrix of Fig. 1. Increasing α* (greater contribution of Aβ42) generally results in lower cutoff values, as cognitive worsening (higher absolute ADAS-Cog scores) is amplified by Aβ42 contribution. This is equivalent to decreasing the gray box in the Aβ load matrix of Fig. 1.

Interestingly, when δ = 0 (i.e., assuming no beneficial effect of Aβ40), the lowest ADAS-Cog value in this MCI population with Aβ + load is about 14 at a cutoff value of 2 units, increasing to 17 at a cutoff value of 15 units. In this case, there are no values that would match the observed clinical outcomes on the ADAS-Cog (Fig. 2). We will address this issue in detail in a later section.

For higher values of *x*_0_ (resulting in more matrix points with beneficial outcomes on glutamate neurotransmission), this cutoff value increases. In order to have a large dynamic range for Aβ modulation to work with, we fix *x*_0_ (position of maximal beneficial effect of Aβ40) at 2 (but see also later). Therefore, the best parameter sets in accordance with the observed ADAS-Cog difference suggests a value of 3 units in our computer model that corresponds to a SUVR of 1.34 [33] when measured with florbetapir.

This cutoff value of 3 units in our model also defines the amyloid-positive and negative patient subgroups in the subsequent simulations on the effect of scopolamine in MCI patients and APOE genotype in AD patients (see later for a more detailed sensitivity analysis).

### Amyloid-beta and scopolamine-induced cognitive deficit in a model of MCI subjects

This section deals with the impact of Aβ load on cholinergic dysfunction caused by scopolamine treatment in a model of an MCI patient population. A clinical study in unmedicated healthy MCI patients [18] indeed suggests that amyloid load affects the recovery after scopolamine application with a higher sensitivity for Aβ + MCI subjects.

As already mentioned, we simulate an MCI patient population in the model using a 3% decrease in synapse and neuron density in addition to a 30% increase in cholinergic tone [25]¸ derived the dose–response of scopolamine for low (Aβ–) and high (Aβ+) Aβ loads based on the cutoff value of 3 units as defined earlier in the cognitive network model, and extrapolated the results to the 2-back working memory paradigm using the calibration derived earlier on the 2-back working memory model [34].

**Fig. 3:**
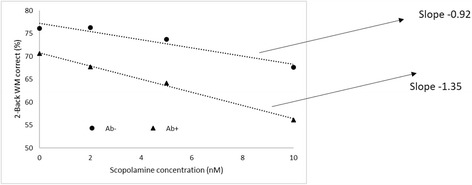
Dose–response of scopolamine-mediated cognitive deficits in a MCI population with low and high Aβ load corresponding to 3 units of Aβ load in our model for δ = 0.025, α = 0.002 and α* = 0.002. Slopes for the Aβ– and Aβ + conditions are −1.08 and −1.36% correct answers/nM scopolamine, respectively, for a 2-back working memory (WM) test. Model outcome suggests that higher Aβ load makes the system more sensitive to scopolamine; that is, a greater deficit for the same scopolamine dose that would correspond to the clinically observed slower recovery after scopolamine in MCI subjects. Pharmacodynamic interaction with Aβ can have important consequences as standard of care for AD patients often includes procholinergic compounds. Aβ amyloid-beta, Aβ– subjects with *x* ≤ 2 and *y* ≤ 2, Aβ + subjects with *x* > 2 and *y* > 2

Figure 3 shows the dose–response of scopolamine for Aβ– and Aβ + MCI patients for δ = 0.025, α = 0.002 and α* = 0.002. Because the slope for Aβ + is greater in magnitude than for Aβ–, under these conditions the system becomes more sensitive to scopolamine under the positive amyloid load condition. The case of a more sensitive dose–response for scopolamine in Aβ + MCI subjects can be achieved for δ ≥ 0.02 and for α and α* ≤ 0.0025. The absolute values of the slopes increase with higher coupling α between Aβ and Glu neurotransmission, but the relative difference remains constant; this is likely due to the indirect effect of α7 nAChR on glutamate levels and the balance with α4β2 nAChR-mediated GABA tone due to scopolamine. Indeed, scopolamine indirectly affects nicotinic receptor activation levels through its inhibition of the presynaptic M_2_ muscarinic autoreceptor that elevates synaptic ACh levels. Increasing α* (greater contribution of Aβ42-mediated decrease in Glu neurotransmission) enhances the differences in slopes (data not shown).

Again when δ = 0 (i.e., no beneficial effect of Aβ40), slopes for the Aβ– patients (x ≤ 2 and *y* ≤ 2) are greater (–1.54% accuracy loss per nM scopolamine dose) than for Aβ + subjects (−1.42% accuracy loss per nM scopolamine). A more detailed sensitivity analysis will be reported in a later section.

### Impact of APOE genotype on amyloid-beta-mediated cognitive trajectory

We subsequently studied the impact of the APOE genotype on the cognitive trajectory of a ‘mild-to-moderate AD’ virtual patient population with different baseline Aβ loads over a total of 78 weeks. Again, we assume a linear increase of the different Aβ peptides at 0.5, 1 and 1.5 units/13 weeks, respectively for APOE4^−/−^, APOE4^+/−^ and APOE4^+/+^ (see Methods).

**Fig. 4:**
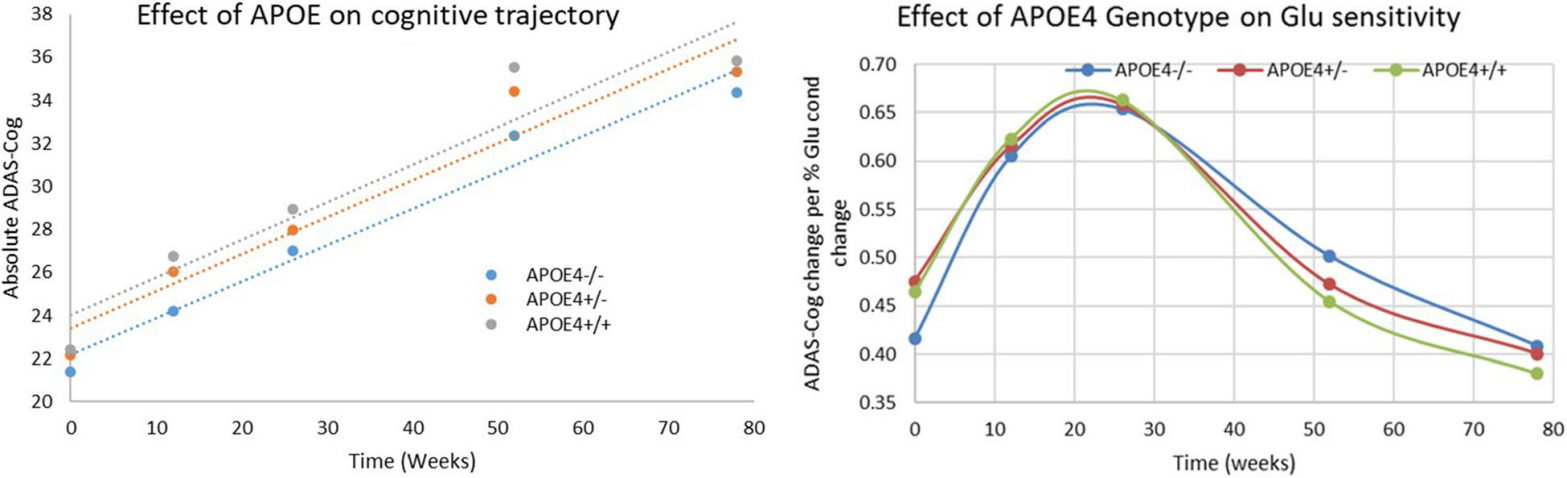
**a** Simulated outcome of APOE genotype on changes in ADAS-Cog for placebo patients with mild Aβ starting load (4 units) over 78 weeks with APOE4^+/+^ genotype implemented as having lower synaptic density (−20%) and lower clearance of Aβ. Platform outcome suggests that under these conditions APOE merely drives the baseline difference but does not affect disease progression. **b** Slopes of glutamate coupling (expressed as change in ADAS-Cog per % change in glutamate neurotransmission) as a function of disease progression (measured from start of a trial in a mild-to-moderate AD population) and APOE4 genotype. In the pathological state, defined by a range between 12 and 34 weeks into the trial, the system is maximally sensitive to NMDA conductance changes and therefore to Aβ-mediated effects. Note that while the APOE4^+/+^ genotype has a greater sensitivity to Aβ load changes in the early pathology stages, the trend is reversed at more extensive pathology. However, with greater Aβ loads seen at later pathology stages, the range available to see an effect is reduced. This would also suggest that changes in amyloid levels from therapeutic interventions tend to have a maximal effect at 10–35 weeks in a trial with mild-to-moderate AD patients. ADAS-Cog Alzheimer Disease Assessment Scale, cognitive subscale, APOE apolipoprotein E, Glu glutamate

Figure 4a illustrates the cognitive trajectory of ‘virtual patients’ with the APOE genotype for patients with a ‘mild baseline’ Aβ + load (4units which is  above the cutoff level of 3). The results suggest that in these conditions the progression rate of the ADAS-Cog is relatively independent of the APOE genotype.

We subsequently tested the variation of the three APOE genotype-specific slopes of the cognitive trajectory as a function of different Aβ loads. Generally, the trajectories are more parallel (smaller variation) for APOE AD patients with higher Aβ + loads. This illustrates the complex pharmacodynamics relationship between synapse density, Aβ clearance (mediated by APOE4 genotype) and the differential effect of both peptides on glutamate and nicotinic neurotransmission.

Figure 4b illustrates the effect of glutamate conductance changes of the excitatory–excitatory synapses on the ADAS-Cog for different APOE genotypes. This is proportional to changes in Aβ load when isolated to the glutamatergic effects (i.e., modifying α and α* for a fixed *x*, *y* or increasing *x* and *y* to show the effects of α and α*). The system has a greater sensitivity at earlier time points (i.e., 12 and 26 weeks) compared to 52 and 78 weeks, with the slopes at 0 weeks somewhat intermediate with about 0.65 points on the ADAS-Cog per % change in Glu conductance at 12 and 26 weeks versus 0.4 points at 52 and 78 weeks. This suggests that the clinical effects of removing Aβ oligomers have a stronger effect earlier instead of later. There is a complex effect of the APOE4 genotype load in that APOE4^+/+^ subjects have different slopes compared to the APOE4^−/−^ subjects, which are 11%, 3% and 1% greater at 0, 12 and 26 weeks, and 8% and 10% smaller at 52 and 78 weeks.

When δ = 0 (i.e., no beneficial effect of Aβ40), the differences between the slopes of cognitive decline for APOE4^+/+^ genotype vs APOE4^−/−^ combinations are in the range of 10% (see also Sensitivity analysis).

### Sensitivity analysis: arguments for an Aβ40-mediated neurostimulatory effect in human subjects

A key assumption with a substantial implication is the presence of a neurostimulatory effect of short Aβ fragments in a specific dose range. As mentioned earlier, with δ = 0 we were unable to reproduce both the observed difference between MCI Aβ + and MCI Aβ– on the ADAS-Cog or the greater sensitivity of the scopolamine dose–response in MCI Aβ + subjects.

We performed a systematic sensitivity analysis for parameters α, α*, β and δ for a fixed value of *x*_0_ = 2 that would generate a model fulfilling all of the following three conditions as outlined in the previous three results sections: an outcome of 8.5 ± 1 points on the ADAS-Cog in MCI Aβ– patients and of 10.7 ± 1 points for Aβ + MCI subjects with a cutoff value < 4; a greater sensitivity to scopolamine in MCI Aβ + subjects; and a difference of at most 10% between cognitive trajectory slopes for APOE4^+/+^, APOE4^+/−^ and APOE4^−/−^ with the additional conditions that the APOE4^+/+^ genotype is at least 1.5 points worse than the APOE4^−/−^ at time 0 weeks.

**Fig. 5:**
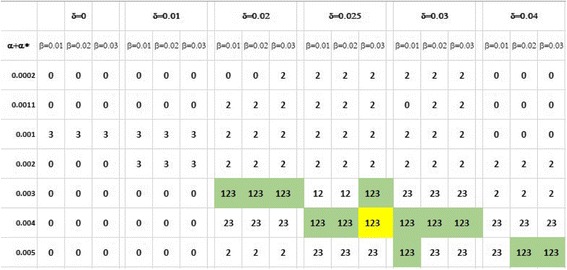
Systematic sensitivity analysis of model outcome for all key parameters of the model with *x*_0_ fixed at 2. Each cell lists which of three conditions are met: Condition 1, outcome of 8.5 ± 1 points on ADAS-Cog in MCI Aβ– patients and 10.7 ± 1 points for Aβ + MCI subjects with cutoff value < 4; Condition 2, greater sensitivity to scopolamine in MCI Aβ + subjects; Condition 3, maximum difference of 10% between cognitive trajectory slopes for APOE4^+/+^, APOE4^+/−^ and APOE4^−/−^ with the additional requirement that APOE4^+/+^ genotype is at least 1.5 points worse than APOE4^−/−^ at time 0 weeks. Only for some cells with δ > 0.02 and (α + α*) > 0.003 are all three conditions are met simultaneously. Note that for δ = 0 (i.e., no protective effect of the short Aβ form), no case exists where all three conditions are met simultaneously

Figure 5 shows that all three conditions are met only when δ > 0.02 and for α + α* > 0.003. We furthermore explored the parameter space for α, α*, β and δ for a fixed value of *x*_0_ = 4 (i.e., greater than the Aβ+ cutoff value of 3). In this case, parameter values for all three conditions to be met shift to higher values, such that δ > 0.03 and α + α* > 0.004 with a slight preference for higher β values.

We use the values *x*_0_ = 2, δ = 0.025, β = 0.03, α = 0.002 and α* = 0.002 for the analysis in the next section.

### Therapeutic amyloid-beta interventions on anticipated clinical outcome

In this section, we simulate the effect of changes in levels of Aβ oligomers with therapeutic interventions such as solanezumab, BACE inhibitors (BACE-I) and gamma-secretase inhibitors (GSI) on the anticipated ADAS-Cog outcome. We introduce real clinical target engagement data (i.e., changes in soluble Aβ40 and Aβ42 levels) from the Expedition I and II trials with solanezumab [35], the gamma-secretase inhibitor semagacestat [36] and the BACE inhibitor verubecestat [37]. Both the BACE inhibitor (80–90% reduction in Aβ40 and 60–80% for Aβ42) and the gamma-secretase inhibitor (30–50% reduction for Aβ40 and 15–30% for Ab42) affect Aβ40 more than Aβ42, while the inverse is true for solanezumab (5–10% inhibition of Aβ40 and 30–50% reduction of Aβ42). These measurements mostly report monomer Aβ forms with a small contribution from dimers and trimers. Reduction in monomer levels likely leads to reduced oligomeric forms, as they are formed from combinations of monomers although a smaller fraction might come from breakdown of larger aggregates. Therefore, here we assume that these changes in clinically measured soluble Aβ levels reflect the level of oligomeric central Aβ and are present for the whole duration of the clinical trial with steady-state kinetics achieved relatively rapidly.

We simulated the anticipated cognitive trajectory for two different baseline Aβ loads as defined by amyloid imaging and converted to ‘units’ in the computer model outlined earlier. Placebo patients increase their Aβ load by 1 unit/13 weeks and their cognition at 78 weeks worsens by 8.5–10 points on the ADAS-Cog dependent upon their amyloid baseline status (from calibration using historical clinical trials [16]). Note that the ADAS-Cog worsening is for a very large part due to a continuous loss of synapses and neuronal cells caused by nonamyloid toxicity. Patients on the active drugs have a proportionally lower Aβ40 and Aβ42 increase according to their biomarker change; for instance, in the case of a low dose of GSI, a 40% reduction in Aβ40 and a 20% reduction in Aβ42 corresponds to an increase of 0.6 units along the Aβ40 axis and 0.80 units along the Aβ42 axis/13 weeks. Furthermore, we sample a 3 × 3 matrix around the Aβ load at that particular time point; that is, for a patient at Aβ load of *x* = 4 and *y* = 4 we take the average of the 3 × 3 matrix, 3 ≤ *x* ≤ 5 and 3 ≤ *y* ≤ 5. Treatment with active drugs often results in fractional units (see earlier); in that case we take a weighted average so that the mass average of the Aβ load corresponds to the actual load. For instance, in the earlier example, while placebo subjects move to point 5 from 0 to 12 weeks with nine equal weightings between 4 and 6, the outcome for a subject on low-dose BACE-I (reducing Aβ40 by 80% and Aβ42 by 60%) is calculated using a weighting factor of 0.73, 0.33 and −0.06 for the Aβ40 units of 4, 5 and 6; similarly, for Aβ42 the weighting factors are 0.64, 0.33 and 0.03.

**Fig. 6:**
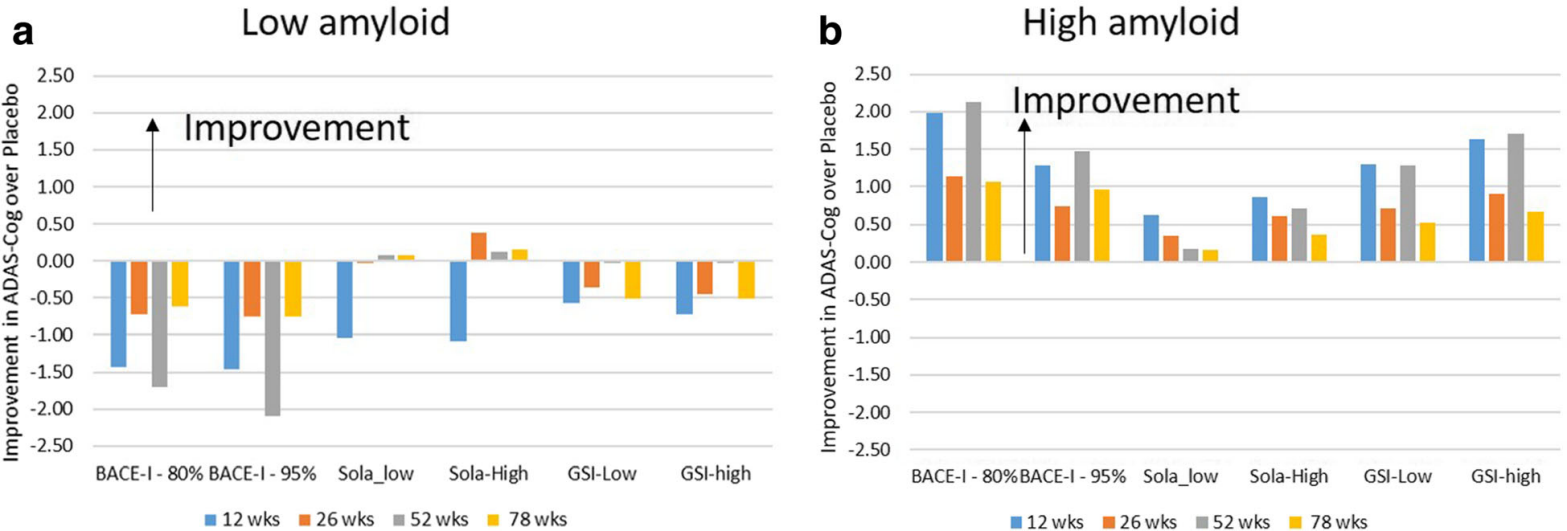
**a** Simulated clinical outcomes in ADAS-Cog in a 78-week trial with mild-to-moderate AD patients with low amyloid baseline (amyloid load negative) after BACE inhibition (verubecestat), gamma-secretase inhibition (semagacestat) and solanezumab (Sola) antibody. Differences with placebo values shown. BACE-I results in a substantial worsening over the whole trial duration, with a somewhat lower negative effect of GSI and almost no effect of solanezumab. **b** Simulated clinical outcomes in ADAS-Cog in a 78-week trial with mild-to-moderate AD patients with medium to high amyloid baseline (amyloid load positive) after BACE inhibition (verubecestat), gamma-secretase inhibition (semagacestat) and solanezumab antibody. Differences with placebo values are shown. BACE-I improves cognition, with substantial benefit (1–2 points) at longer time points, while GSI has a smaller dose-dependent response (1–1.5 points). Note that higher BACE inhibition is less beneficial. Solanezumab, on the contrary, has a modest dose-dependent clinical benefit (0.5–1 points) ADAS-Cog Alzheimer Disease Assessment Scale, cognitive subscale, BACE-I BACE inhibitor, GSI gamma-secretase inhibitor, wks weeks

For patients starting at a negative or low  Aβ load (SUVR < 1.1 or < 3 units in the model), BACE-I tends to worsen cognition over the whole trial duration (Fig. 6a) with losses of 1–2 points on the ADAS-Cog, GSI worsens outcome by 0.4–0.8 points, while solanezumab’s effect ranges from 1 point worsening to 0.5 point improvement. In contrast, for a patient starting at a high Aβ amyloid load of 8, corresponding to a SUVR well above 1.1, almost all interventions improve cognition (Fig. 6b) and most significantly at later time points. While solanezumab and GSI outperform placebo by about 1 point on the ADAS-Cog, BACE-I shows the biggest benefit nearing 2 points. This difference with placebo increases with higher baseline Aβ load for all three interventions (data not shown), but the effects saturate between 1.5 and 2.0 points on the ADAS-Cog. Note that the effects are generally dose dependent for both improvement and worsening for solanezumab and GSI, but not for BACE-I.

These effects, both in the negative and positive directions for all three compounds, become more pronounced when *x*_0_ = 4; that is, when the position of optimal Aβ40-mediated protection is higher than the cutoff value. Therefore, comparing the model outcomes with actual clinical data on patients from the different trials with their individual amyloid level might further constrain the model parameter x_0_.

The data suggest a ‘sweet spot’ for modulating Aβ40. Starting from low baseline levels of Aβ, reducing Aβ40 has a negative effect because the stimulatory Aβ40 effect that dominates the negative Aβ42 effect is lost. Because solanezumab does not affect Aβ40 as much, it has a modest beneficial effect on cognition relatively independent of the baseline amyloid load.

These results have been simulated in a situation without standard-of-care medication. Pharmacodynamic interactions with Aβ can be expected as donepezil, by extending the half-life of ACh, affects both α_4_β_2_ and α_7_ nAChR. Donepezil addition worsens the cognitive readout by 0.2 points for all interventions for low Aβ starting levels, but slightly improves the outcome for BACE inhibition with about 0.5 points for high Aβ baseline levels (data not shown). This illustrates the complex nonlinear interaction between neurostimulatory vs neuroinhibitory properties of the different amyloid forms.

## Discussion

This report describes a computer-based quantitative systems pharmacology model of the impact of low-order Aβ40 and Aβ42 aggregates with their differential neurophysiological effects on glutamatergic and nicotinic neurotransmission in a cognitive calibrated ADAS-Cog model. Great care is taken to constrain the parameters with three independent clinical datasets. To our knowledge this is the first time that the biological effects of Aβ peptides on processes relevant for cognitive outcome are explicitly modeled to simulate the outcome for different therapeutic amyloid modulating interventions, starting from their respective pharmacodynamic changes on Aβ levels.

The model assumes that small-order oligomers (dimers, trimers) but not aggregated forms affect synaptic plasticity and neuronal function [38, 39]. A number of biological targets for oligomeric Aβ have been identified (for a review see [40]). We chose here only two, NMDA-R [41] and α7 nAChR [24], as they are directly linked to cellular excitability and action potentials which in turn drive cognitive changes. Later versions could incorporate other targets such as the effect of Aβ on A-type K^+^ channels [42] and the upregulation of 5-HT_1A_ by Aβ40, but not Aβ42 [43] and the neuronal calcium dysregulation [44], to obtain a more comprehensive view of Aβ effects on neuronal dynamics.

Experimental studies suggest that dimers, trimers, tetramers and octamers all affect cognition [45, 46]. Furthermore, Aβ could interact with the glutamatergic systems in a number of ways. Studies in transgene mouse models and AD brains have found that plaque load is correlated with lower expressions of VGLUT1 [47]. The model further assumes linear effects of therapeutic interventions by amyloid-lowering agents. While this might be appropriate for BACE inhibition or GSI because blocking the synthesis of Aβ has a direct effect on the formation of oligomers, it might not be the case for antibody-related interventions, as they might affect various components in the aggregation pathway. In this case, a more detailed mechanistic model of Aβ aggregation and the impact of different affinities of the antibody might be needed [48, 49].

Here we implement the differential interaction on the paired pulse amplification or reduction in an in-vitro slice electrophysiology [5, 50] by introducing an Aβ species-specific effect on excitatory–excitatory NMDA conductance. A more detailed computational model explores a different mechanism of Aβ oligomer-induced NMDA dysfunction [51].

The simulations suggest that the contribution of Aβ-mediated glutamate conductance changes on cognitive outcome tends to decrease with more advanced pathology. Also, the effect of glutamate conductance change on the system outcome is more sensitive to APOE4 load at earlier stages of the pathology, but reverses at more severe AD pathology (i.e., greater effect in APOE4^–/–^ carriers). There is indeed evidence that APOE4-associated effects play a more important role early in the disease; that is, by determining the age of onset [52], rather than the progression of the disease [19, 32, 53]. This also suggests that the effect of APOE4 genotype on Aβ clearance and synaptic dysfunction becomes less important as the disease progresses.

Importantly, when removing the neuroprotective nature of Aβ40 on Glu neurotransmission (by fixing the maximal effect δ at 0), the model generates outcomes that are incompatible with clinical observations such as the ADAS-Cog average values for MCI subjects, the differential sensitivity of Aβ– vs Aβ + MCI subjects to scopolamine and the impact of APOE on slopes of cognitive worsening in untreated AD patients. This suggests that in the human AD condition, shorter Aβ isoforms (here represented by Aβ40) might be beneficial which might explain the limited clinical response for the different amyloid-modulating therapeutic interventions.

With the parameter values that reproduce the clinical observations on APOE trajectory, absolute ADAS-Cog values for Aβ + and Aβ– MCI subjects and scopolamine effects in MCI, the computer model suggests that the modest effects of various amyloid-modulating interventions on the ADAS-Cog are highly dependent upon the starting amyloid load. For patients starting out with low baseline Aβ load, reducing Aβ40 and Aβ42 indiscriminately worsens cognition as the stimulatory effects of Aβ40 outweighs the inhibitory effects of Aβ42. In contrast, subjects starting out with high amyloid baseline all improve when amyloid is reduced, but even then the maximal benefit is around 0.8 points for solanezumab (in line with clinical data [54]) while optimal doses of BACE-I can result in benefits of up to 2 points. The model also explains why many of the amyloid-modulating agents work well in transgene animal models where a high level of amyloid is artificially generated to accelerate the pathology.

For studies relying on clinical diagnosis and not using enrichment based on amyloid load, it has been suggested that up to 1/3 of ‘Alzheimer’ patients had an Aβ level below the SUVR cutoff value, especially in the non-APOE4 carriers as found in the phase III bapineuzumab trials [55]. Therefore, if the trial does not specifically enrich for amyloid-positive subjects, the model suggests essentially that group average would not be different from placebo.

A major unresolved issue is the significant clinical worsening of the GSI semagacestat [56], where a 1.4 point worsening on the ADAS-Cog has been detected. The model with the parameter settings constrained by the clinical data predicts only a modest worsening (0.80 points) for subjects with low Aβ load. This is likely due to the modest level of target engagement as BACE inhibition at 95% can easily generate a 2 point worsening in subjects with low Aβ load. The way we bring in the pharmacodynamic effect of GSI on low-order Aβ aggregates is likely incomplete and a more complex mechanism needs to be developed, such as the Aβ rebound [57] with GSI but not gamma-secretase modulators, more complex enzymatic biphasic kinetics [58] or even its pseudoinhibition character [59].

The model also suggests that the impact of Aβ on α7 nAChR is much more limited than the effect on the glutamatergic system. The relation between Aβ and α7 nAChR is complex. In a study of 29 elderly clergy, irrespective of cognitive status, higher amyloid plaque load was associated with elevated expression of α7 nAChR [60], which could be explained by a compensatory upregulation after chronic inhibition [61]. The relevant coupling parameters between α7 nAChR activation and the impact on presynaptic glutamate release are derived from the neurophysiology of AChE-I and have been calibrated with ADAS-Cog outcome. The limited contribution of this process to global cognition would be in line with the modest clinical results of α7 nAChR modulators [62] in general and ABT-107 and TC-5619 in AD. The relatively strong positive effect observed with enicicline or EVP-6124 in AD [63] might also be due to the additional 5-HT3 antagonism pharmacology of this clinical candidate [64].

There is a vast literature about the effects of APOE on Aβ load, with the consensus that APOE4 carriers have higher amyloid load [65], probably because of a differential clearance of Aβ [66]. We implemented this effect by setting the increase in Aβ40 and Aβ42 load to 1.5 units/13 weeks, 1 unit/week and 0.5 units/13 weeks for the APOE4^+/+^, APOE4^+/−^ and APOE4^−/−^ genotype, respectively. Interestingly, other genotypes such as the C667T SNP of the LRP-1 gene might additionally affect Aβ load [21]. In addition, we assumed a 20% increase in synaptic density for APOE4^−/−^ carriers and a 20% decrease for APOE4^+/+^ carriers as compared to the ‘wild-type’ APOE4^+/−^ genotype [31]. These results are mostly obtained in transgene animal models and are not found in postmortem AD brain [67], although findings in human studies might reflect final stages of the disease and not fully take into account the differential vulnerability of synapses in different APOE genotypes.

The model simulations suggest that the biggest slopes of ADAS-Cog deterioration as a function of the effect of Aβ on glutamate neurotransmission arises for an interval of 10–35 weeks. Because removal of Aβ oligomers by vaccination strategies or enzyme inhibition would improve glutamate conductance, this suggests that the biggest impact of these therapeutic interventions might be in that timeframe. Interestingly the biggest improvement in the highest dose of a small aducanumab trial was observed between 26 and 52 weeks [68].

The effect of Aβ load on cognitive performance after scopolamine outcome in MCI patients suggests that the system is more sensitive to muscarinic receptor antagonism at higher Aβ baseline, in line with clinical data [18]. Further sensitivity analysis shows that this effect is present for a range of coupling parameters, with a slightly bigger impact of the coupling with α7 nAChR. This is probably due to the fact that scopolamine is a strong presynaptic M2 mAChR autoreceptor inhibitor, leading to higher levels of ACh and indirectly affecting the activity of nicotinic ACh receptors. Whether this has important consequences for possible pharmacodynamic interactions between cholinergic medications such as AChE-I and amyloid-modulating interventions needs to be studied in more detail. Further expansion of this model to take into account the effect of neuronal activity on APP synthesis [69] can further provide insights into the unexpected pharmacodynamic effects of procholinergic treatment.

This model makes a number of predictions which could easily be tested based on clinical data from industry-sponsored trials, in which we anticipate that clinical response would be very different between AD patients with low vs high Aβ load at the start of the trial. We believe it is absolutely essential to test the predictive validity of this model, as the consequences are far reaching. In fact, the model when properly validated would strongly discourage testing amyloid-lowering agents in presymptomatic AD and elderly controls, even with APOE4^+/+^ genotypes, if they do not already have high amyloid load. A number of clinical studies with amyloid-lowering agents have already started using either passive [70] or active vaccination [71] based on the premise of stopping the ‘neurotoxic’ effects of Aβ before they start. As the model points out, Aβ physiology is considerably more complex and some Aβ forms might provide beneficial effects, suggesting that early interventions would actually worsen cognitive outcome.

There are a large number of limitations in the model. A key assumption in this model is the lack of direct neurotoxicity from Aβ in the human brain. There are no data on the direct relation between amyloid accumulation and brain volume loss, as many other processes (which we do not model) such as neuroinflammation and tau pathology triggered by higher Aβ levels might drive cellular neurotoxicity. Note that the QSP model already indirectly assumes a linear loss of synapses and neurons over time [16]. Many APP overexpressing mouse models with high levels of plaque load do not show overt neurotoxicity or synapse loss. For instance, in a traumatic brain injury model of controlled impact, the gamma-secretase inhibitor semagacestat reduces Aβ load, but not synaptic density loss [72]. Likewise, region-of-interest imaging in cognitively normal subjects shows differences in hypometabolism but not atrophy in Aβ + vs Aβ– subjects [73] and tau PET imaging is correlated much more with cortical atrophy than Aβ load [74].

Another challenge is to link the calculated level of aggregated Aβ forms in the computer model to some clinical experimental amyloid imaging load, such as a standardized uptake value ratio (SUVR) with a PET imaging tracer. In this study, we defined a relationship between the SUVR and ‘units’ of Aβ load based on cognitive readouts in Aβ + and Aβ– MCI subjects. While this allowed us to link the SUVR threshold to a cutoff value in units of Aβ load, we assumed a linear relationship above and below this cutoff value. However, to improve this simplistic assumption, we need to bring in the affinity of the tracers for the various forms of Aβ and expand the model to take into account a spatial component. Both a three-compartment model with neurons and astrocytes, the perivascular system and cerebral vasculature [75] and a detailed spatiotemporal model of cerebral vasculature and perivascular glymphatic clearance [76] could serve as a good starting point. This would enable us to better constrain the Aβ progression over time and provide readouts that are better correlated with clinical observations. Another interesting issue is the relationship between SUVR measurement and the optimal Aβ40 level for its stimulatory effect, as this can define better criteria for patient selection. We showed that increasing the optimal Aβ40 level would amplify any negative outcomes of indiscriminate Aβ lowering, but this can only be determined from comparing the outcomes of the model with actual clinical studies.

Furthermore, we only simulate the dynamics of two forms of Aβ peptides, while there are presumably many more in the AD brain. For instance, the SILK studies also report on the dynamics of Aβ1–38 [3, 77]. The same reasoning applies to the recently discovered and potentially important η-amyloid [78]. Once more detailed data on the neurophysiological effects of these amyloid species become available they can be included in the model. This version of the model essentially considers two different generic Aβ peptides with differential neurophysiology and can therefore explore the rationale for gamma-secretase modulators that aim to reduce the level of neuroinhibitory long Aβ isoforms while increasing the level of shorter neurostimulatory Aβ isoforms [79].

Moreover, the model does not address other pathological conditions that drive the cognitive deficit in the absence of amyloid, such as vascular or tau pathology. In principle, given enough information on the biology of these processes, QSP modeling could address these additional pathways, although this is beyond the scope of the present report.

In summary, this report is a first attempt to relate neurophysiological effects of Aβ peptides to cognitive readout in a comprehensive humanized computer modeling platform. The presence of an Aβ40-mediated neurostimulatory process in the human Alzheimer brain is supported by an extensive sensitivity analysis showing that this is necessary to explain three independent clinical datasets. Ideally as a prospective validation, the outcomes of this model should be compared to the changes in cognition for individual patients in a number of past and ongoing clinical trials, based on an estimate of their Aβ load and comedications. Once properly validated, such approaches could be extremely helpful for supporting clinical projects in amyloid modulation, including combination therapies of vaccination strategies, BACE inhibitors and gamma-secretase modulators, and providing guidance to identify a responder patient population.

## Conclusion

Using advanced mechanism-based computer modeling we integrated preclinical and clinical data to generate a model for the effects of Aβ changes on cognition in Alzheimer patients. A biphasic dose–response with a neurostimulatory dose range for the shorter Aβ forms was necessary to explain three different clinical datasets.

When this model was applied to reported pharmacodynamic changes with three Aβ therapies in clinical trials, the model predicted a cognitive improvement only for patients with high Aβ load, but a worsening of cognition for patients with zero or low Aβ load. If these predictions can be confirmed by post-hoc analysis of failed clinical trials, this questions the rationale for testing Aβ therapies in presymptomatic subjects with low or zero amyloid load.

## Abbreviations

AChE-I: Acetylcholinesterase inhibitor
AD: Alzheimer’s disease
ADAS-Cog: Alzheimer Disease Assessment Scale, cognitive subscale
APOE: Apolipoprotein E
APP: Amyloid precursor protein
Aβ40: Amyloid-beta 1–40
Aβ42: Amyloid-beta 1–42
mAChR: Muscarinic acetylcholine receptor
MCI: Minimal cognitive impairment
MMSE: Mini-Mental State Examination
nAChR: Nicotinic acetylcholine receptor
NMDA: *N*-methyl-d-aspartate
QSP: Quantitative systems pharmacology
SILK: Stable isotope labeling kinetics

## References

[1] Hardy JA, Higgins GA (1992). Alzheimer’s disease: the amyloid cascade hypothesis. Science.

[2] Karran E, Hardy J (2014). A critique of the drug discovery and phase 3 clinical programs targeting the amyloid hypothesis for Alzheimer disease. Ann Neurol.

[3] Mawuenyega KG, Kasten T, Sigurdson W, Bateman RJ (2013). Amyloid-beta isoform metabolism quantitation by stable isotope-labeled kinetics. Anal Biochem.

[4] Huang Y, Potter R, Sigurdson W, Santacruz A, Shih S, Ju YE, Kasten T, Morris JC, Mintun M, Duntley S (2012). Effects of age and amyloid deposition on Abeta dynamics in the human central nervous system. Arch Neurol.

[5] Wang Y, Zhou TH, Zhi Z, Barakat A, Hlatky L, Querfurth H (2013). Multiple effects of beta-amyloid on single excitatory synaptic connections in the PFC. Front Cell Neurosci..

[6] Fogel H, Frere S, Segev O, Bharill S, Shapira I, Gazit N, O’Malley T, Slomowitz E, Berdichevsky Y, Walsh DM (2014). APP homodimers transduce an amyloid-beta-mediated increase in release probability at excitatory synapses. Cell Rep.

[7] Abramov E, Dolev I, Fogel H, Ciccotosto GD, Ruff E, Slutsky I (2009). Amyloid-beta as a positive endogenous regulator of release probability at hippocampal synapses. Nat Neurosci.

[8] Sengupta U, Nilson AN, Kayed R (2016). The role of amyloid-beta oligomers in toxicity, propagation, and immunotherapy. EBioMed..

[9] Geerts H, Spiros A, Roberts P, Carr R (2013). Quantitative systems pharmacology as an extension of PK/PD modeling in CNS research and development. J Pharmacokinet Pharmacodyn.

[10] Nyman E, Rozendaal YJ, Helmlinger G, Hamren B, Kjellsson MC, Stralfors P, van Riel NA, Gennemark P, Cedersund G (2016). Requirements for multi-level systems pharmacology models to reach end-usage: the case of type 2 diabetes. Interface Focus.

[11] Geerts H, Spiros A, Roberts P, Twyman R, Alphs L, Grace AA (2012). Blinded prospective evaluation of computer-based mechanistic schizophrenia disease model for predicting drug response. PLoS One.

[12] Nicholas T, Sridhar D, Claire L, David R, Tracey R, Phil I, Carolyn R, Robert C, Patrick R, Athan S, Hugo G (2013). Systems pharmacology modeling in neuroscience: prediction and outcome of PF-04995274, a 5HT4 partial agonist, in a clinical scopolamine impairment trial. Advances Alzheimer’s Dis.

[13] Liu J, Ogden A, Comery TA, Spiros A, Roberts P, Geerts H (2014). Prediction of Efficacy of Vabicaserin, a 5-HT2C agonist, for the treatment of schizophrenia using a quantitative systems pharmacology model. CPT Pharmacometrics Syst Pharmacol..

[14] Peterson MC, Riggs MM (2015). FDA advisory meeting clinical pharmacology review utilizes a quantitative systems pharmacology (QSP) model: a watershed moment?. CPT Pharmacometrics Syst Pharmacol.

[15] Geerts H (2009). Of mice and men: bridging the translational disconnect in CNS drug discovery. CNS Drugs.

[16] Roberts PD, Spiros A, Geerts H (2012). Simulations of symptomatic treatments for Alzheimer’s disease: computational analysis of pathology and mechanisms of drug action. Alzheimers Res Ther.

[17] Doraiswamy PM, Sperling RA, Johnson K, Reiman EM, Wong TZ, Sabbagh MN, Sadowsky CH, Fleisher AS, Carpenter A, Joshi AD (2014). Florbetapir F 18 amyloid PET and 36-month cognitive decline: a prospective multicenter study. Mol Psychiatry.

[18] Lim YY, Maruff P, Schindler R, Ott BR, Salloway S, Yoo DC, Noto RB, Santos CY, Snyder PJ (2015). Disruption of cholinergic neurotransmission exacerbates Abeta-related cognitive impairment in preclinical Alzheimer’s disease. Neurobiol Aging.

[19] Samtani AMXS, Russu A, Adedokun O, Lu M, Ito K, Corrigan B, Raje S, Brashear R, Styren S, Hu C (2015). Alzheimer’s Disease Assessment Scale-cognitive 11 item progression model in mild-to-moderate Alzheimer’s disease trials of bapineuzumb. Alzheimers Dement..

[20] Williams GV, Goldman-Rakic PS (1995). Modulation of memory fields by dopamine D1 receptors in prefrontal cortex. Nature.

[21] Grimmer T, Goldhardt O, Guo LH, Yousefi BH, Forster S, Drzezga A, Sorg C, Alexopoulos P, Forstl H, Kurz A (2014). LRP-1 polymorphism is associated with global and regional amyloid load in Alzheimer’s disease in humans in-vivo. NeuroImage Clin..

[22] Hines ML, Carnevale NT (1997). The NEURON simulation environment. Neural Comput.

[23] Davies P, Maloney AJ (1976). Selective loss of central cholinergic neurons in Alzheimer’s disease. Lancet.

[24] Puzzo D, Arancio O (2013). Amyloid-beta peptide: Dr. Jekyll or Mr. Hyde?. J Alzheimers Dis.

[25] Ikonomovic MD, Mufson EJ, Wuu J, Cochran EJ, Bennett DA, DeKosky ST (2003). Cholinergic plasticity in hippocampus of individuals with mild cognitive impairment: correlation with Alzheimer’s neuropathology. J Alzheimers Dis.

[26] Athan Spiros HG (2012). A quantitative way to estimate clinical off-target effects for human membrane brain targets in CNS Research and Development. J Exp Pharmacol..

[27] Spiros A, Carr R, Geerts H (2010). Not all partial dopamine D(2) receptor agonists are the same in treating schizophrenia. Exploring the effects of bifeprunox and aripiprazole using a computer model of a primate striatal dopaminergic synapse. Neuropsychiatr Dis Treat.

[28] Besnard J, Ruda GF, Setola V, Abecassis K, Rodriguiz RM, Huang XP, Norval S, Sassano MF, Shin AI, Webster LA (2012). Automated design of ligands to polypharmacological profiles. Nature.

[29] Slutsky I, Wess J, Gomeza J, Dudel J, Parnas I, Parnas H (2003). Use of knockout mice reveals involvement of M2-muscarinic receptors in control of the kinetics of acetylcholine release. J Neurophysiol.

[30] Leoni V (2011). The effect of apolipoprotein E (ApoE) genotype on biomarkers of amyloidogenesis, tau pathology and neurodegeneration in Alzheimer’s disease. Clin Chem Lab Med.

[31] Kim J, Yoon H, Basak J (2014). Apolipoprotein E in synaptic plasticity and Alzheimer’s disease: potential cellular and molecular mechanisms. Mol Cells.

[32] Aerssens J, Raeymaekers P, Lilienfeld S, Geerts H, Konings F, Parys W (2001). APOE genotype: no influence on galantamine treatment efficacy nor on rate of decline in Alzheimer’s disease. Dement Geriatr Cogn Disord.

[33] Chiotis K, Carter SF, Farid K, Savitcheva I, Nordberg A (2015). Amyloid PET in European and North American cohorts; and exploring age as a limit to clinical use of amyloid imaging. Eur J Nucl Med Mol Imaging.

[34] Geerts H, Roberts P, Spiros A (2013). A quantitative system pharmacology computer model for cognitive deficits in schizophrenia. CPT Pharmacometrics Syst Pharmacol..

[35] Farlow M, Arnold SE, van Dyck CH, Aisen PS, Snider BJ, Porsteinsson AP, Friedrich S, Dean RA, Gonzales C, Sethuraman G (2012). Safety and biomarker effects of solanezumab in patients with Alzheimer’s disease. Alzheimers Dement.

[36] Doody RS, Raman R, Sperling RA, Seimers E, Sethuraman G, Mohs R, Farlow M, Iwatsubo T, Vellas B, Sun X (2015). Peripheral and central effects of gamma-secretase inhibition by semagacestat in Alzheimer’s disease. Alzheimers Res Ther.

[37] van Maanen EM, van Steeg TJ, Michener MS, Savage MJ, Kennedy ME, Kleijn HJ, Stone JA, Danhof M (2016). Systems pharmacology analysis of the amyloid cascade after beta-secretase inhibition enables the identification of an Abeta42 oligomer pool. J Pharmacol Exp Ther.

[38] Shankar GM, Li S, Mehta TH, Garcia-Munoz A, Shepardson NE, Smith I, Brett FM, Farrell MA, Rowan MJ, Lemere CA (2008). Amyloid-beta protein dimers isolated directly from Alzheimer’s brains impair synaptic plasticity and memory. Nat Med.

[39] Walsh DM, Klyubin I, Fadeeva JV, Cullen WK, Anwyl R, Wolfe MS, Rowan MJ, Selkoe DJ (2002). Naturally secreted oligomers of amyloid beta protein potently inhibit hippocampal long-term potentiation in vivo. Nature.

[40] Goure WF, Krafft GA, Jerecic J, Hefti F (2014). Targeting the proper amyloid-beta neuronal toxins: a path forward for Alzheimer’s disease immunotherapeutics. Alzheimers Res Ther.

[41] Shankar GM, Bloodgood BL, Townsend M, Walsh DM, Selkoe DJ, Sabatini BL (2007). Natural oligomers of the Alzheimer amyloid-beta protein induce reversible synapse loss by modulating an NMDA-type glutamate receptor-dependent signaling pathway. J Neurosci.

[42] Plant LD, Webster NJ, Boyle JP, Ramsden M, Freir DB, Peers C, Pearson HA (2006). Amyloid beta peptide as a physiological modulator of neuronal ‘A’-type K+ current. Neurobiol Aging.

[43] Verdurand M, Chauveau F, Daoust A, Morel AL, Bonnefoi F, Liger F, Berod A, Zimmer L (2016). Differential effects of amyloid-beta 1-40 and 1-42 fibrils on 5-HT1A serotonin receptors in rat brain. Neurobiol Aging..

[44] Lazzari C, Kipanyula MJ, Agostini M, Pozzan T, Fasolato C (2015). Abeta42 oligomers selectively disrupt neuronal calcium release. Neurobiol Aging.

[45] Reed MN, Hofmeister JJ, Jungbauer L, Welzel AT, Yu C, Sherman MA, Lesne S, LaDu MJ, Walsh DM, Ashe KH (2011). Cognitive effects of cell-derived and synthetically derived Abeta oligomers. Neurobiol Aging.

[46] Sherman MA, LaCroix M, Amar F, Larson ME, Forster C, Aguzzi A, Bennett DA, Ramsden M, Lesne SE (2016). Soluble conformers of Abeta and tau alter selective proteins governing axonal transport. J Neurosci.

[47] Rodriguez-Perdigon M, Tordera RM, Gil-Bea FJ, Gerenu G, Ramirez MJ, Solas M (2016). Down-regulation of glutamatergic terminals (VGLUT1) driven by Abeta in Alzheimer’s disease. Hippocampus.

[48] Knowles TP, Waudby CA, Devlin GL, Cohen SI, Aguzzi A, Vendruscolo M, Terentjev EM, Welland ME, Dobson CM (2009). An analytical solution to the kinetics of breakable filament assembly. Science.

[49] Proctor CJ, Boche D, Gray DA, Nicoll JA (2013). Investigating interventions in Alzheimer’s disease with computer simulation models. PLoS One.

[50] Yuan P, Grutzendler J (2016). Attenuation of beta-amyloid deposition and neurotoxicity by chemogenetic modulation of neural activity. J Neurosci.

[51] Liang J, Kulasiri D, Samarasinghe S (2017). Computational investigation of amyloid-beta-induced location- and subunit-specific disturbances of NMDAR at hippocampal dendritic spine in Alzheimer’s disease. PLoS One.

[52] Brayne C, Harrington CR, Wischik CM, Huppert FA, Chi LY, Xuereb JH, O’Connor DW, Paykel ES (1996). Apolipoprotein E genotype in the prediction of cognitive decline and dementia in a prospectively studied elderly population. Dementia.

[53] Diniz LP, Almeida JC, Tortelli V, Vargas Lopes C, Setti-Perdigao P, Stipursky J, Kahn SA, Romao LF, de Miranda J, Alves-Leon SV (2012). Astrocyte-induced synaptogenesis is mediated by transforming growth factor beta signaling through modulation of D-serine levels in cerebral cortex neurons. J Biol Chem.

[54] Doody RS, Farlow M, Aisen PS (2014). Phase 3 trials of solanezumab and bapineuzumab for Alzheimer’s disease. N Engl J Med.

[55] Liu E, Schmidt ME, Margolin R, Sperling R, Koeppe R, Mason NS, Klunk WE, Mathis CA, Salloway S, Fox NC (2015). Amyloid-beta 11C-PiB-PET imaging results from 2 randomized bapineuzumab phase 3 AD trials. Neurology.

[56] Doody RS, Raman R, Farlow M, Iwatsubo T, Vellas B, Joffe S, Kieburtz K, He F, Sun X, Thomas RG (2013). A phase 3 trial of semagacestat for treatment of Alzheimer’s disease. N Engl J Med.

[57] Li T, Huang Y, Jin S, Ye L, Rong N, Yang X, Ding Y, Cheng Z, Zhang J, Wan Z (2012). Gamma-secretase modulators do not induce Abeta-rebound and accumulation of beta-C-terminal fragment. J Neurochem.

[58] Svedruzic ZM, Popovic K, Sendula-Jengic V (2013). Modulators of gamma-secretase activity can facilitate the toxic side-effects and pathogenesis of Alzheimer’s disease. PLoS One.

[59] Tagami S, Yanagida K, Kodama TS, Takami M, Mizuta N, Oyama H, Nishitomi K, Chiu YW, Okamoto T, Ikeuchi T (2017). Semagacestat is a pseudo-inhibitor of gamma-secretase. Cell Rep.

[60] Ikonomovic MD, Wecker L, Abrahamson EE, Wuu J, Counts SE, Ginsberg SD, Mufson EJ, Dekosky ST (2009). Cortical alpha7 nicotinic acetylcholine receptor and beta-amyloid levels in early Alzheimer disease. Arch Neurol.

[61] Jin Y, Tsuchiya A, Kanno T, Nishizaki T (2015). Amyloid-beta peptide increases cell surface localization of alpha7 ACh receptor to protect neurons from amyloid beta-induced damage. Biochem Biophys Res Commun.

[62] Geerts H (2012). alpha7 Nicotinic receptor modulators for cognitive deficits in schizophrenia and Alzheimer’s disease. Expert Opin Investig Drugs.

[63] Deardorff WJ, Shobassy A, Grossberg GT (2015). Safety and clinical effects of EVP-6124 in subjects with Alzheimer’s disease currently or previously receiving an acetylcholinesterase inhibitor medication. Expert Rev Neurother.

[64] Boess FG, De Vry J, Erb C, Flessner T, Hendrix M, Luithle J, Methfessel C, Riedl B, Schnizler K, van der Staay FJ (2007). The novel alpha7 nicotinic acetylcholine receptor agonist N-[(3R)-1-azabicyclo[2.2.2]oct-3-yl]-7-[2-(methoxy)phenyl]-1-benzofuran-2-carboxa mide improves working and recognition memory in rodents. J Pharmacol Exp Ther.

[65] Fouquet M, Besson FL, Gonneaud J, La Joie R, Chetelat G (2014). Imaging brain effects of APOE4 in cognitively normal individuals across the lifespan. Neuropsychol Rev.

[66] Wildsmith KR, Holley M, Savage JC, Skerrett R, Landreth GE (2013). Evidence for impaired amyloid beta clearance in Alzheimer’s disease. Alzheimers Res Ther.

[67] Corey-Bloom J, Tiraboschi P, Hansen LA, Alford M, Schoos B, Sabbagh MN, Masliah E, Thal LJ (2000). E4 allele dosage does not predict cholinergic activity or synapse loss in Alzheimer’s disease. Neurology.

[68] Sevigny J, Chiao P, Bussiere T, Weinreb PH, Williams L, Maier M, Dunstan R, Salloway S, Chen T, Ling Y (2016). The antibody aducanumab reduces Abeta plaques in Alzheimer’s disease. Nature.

[69] Cheng X, Wu J, Geng M, Xiong J (2014). Role of synaptic activity in the regulation of amyloid beta levels in Alzheimer’s disease. Neurobiol Aging.

[70] Sperling RA, Rentz DM, Johnson KA, Karlawish J, Donohue M, Salmon DP, Aisen P (2014). The A4 study: stopping AD before symptoms begin?. Sci Transl Med..

[71] Farlow MR, Andreasen N, Riviere ME, Vostiar I, Vitaliti A, Sovago J, Caputo A, Winblad B, Graf A (2015). Long-term treatment with active Abeta immunotherapy with CAD106 in mild Alzheimer’s disease. Alzheimers Res Ther.

[72] Winston CN, Chellappa D, Wilkins T, Barton DJ, Washington PM, Loane DJ, Zapple DN, Burns MP (2013). Controlled cortical impact results in an extensive loss of dendritic spines that is not mediated by injury-induced amyloid-beta accumulation. J Neurotrauma.

[73] Kljajevic V, Grothe MJ, Ewers M, Teipel S (2014). Distinct pattern of hypometabolism and atrophy in preclinical and predementia Alzheimer’s disease. Neurobiol Aging.

[74] Xia C, Makaretz SJ, Caso C, McGinnis S, Gomperts SN, Sepulcre J, Gomez-Isla T, Hyman BT, Schultz A, Vasdev N (2017). Association of in vivo [18 F]AV-1451 tau PET imaging results with cortical atrophy and symptoms in typical and atypical Alzheimer disease. JAMA Neurol.

[75] Kyrtsos CR, Baras JS (2015). Modeling the role of the glymphatic pathway and cerebral blood vessel properties in Alzheimer’s disease pathogenesis. PLoS One.

[76] Diem AK, Tan M, Bressloff NW, Hawkes C, Morris AW, Weller RO, Carare RO (2016). A simulation model of periarterial clearance of amyloid-beta from the brain. Front Aging Neurosci..

[77] Potter R, Patterson BW, Elbert DL, Ovod V, Kasten T, Sigurdson W, Mawuenyega K, Blazey T, Goate A, Chott R (2013). Increased in vivo amyloid-beta42 production, exchange, and loss in presenilin mutation carriers. Sci Transl Med..

[78] Willem M, Tahirovic S, Busche MA, Ovsepian SV, Chafai M, Kootar S, Hornburg D, Evans LD, Moore S, Daria A (2015). eta-Secretase processing of APP inhibits neuronal activity in the hippocampus. Nature.

[79] Panza F, Frisardi V, Solfrizzi V, Imbimbo BP, Logroscino G, Santamato A, Greco A, Seripa D, Pilotto A (2011). Interacting with gamma-secretase for treating Alzheimer’s disease: from inhibition to modulation. Curr Med Chem.

